# Diagnostic accuracy of artificial intelligence in detection of ovarian cancer—a pilot study

**DOI:** 10.3389/fmed.2026.1729412

**Published:** 2026-02-23

**Authors:** Dipanwita Banerjee, Ashok Sharma, Ekta Dhamija, Sahar Qazi, Sandeep R. Mathur, Neerja Bhatla

**Affiliations:** 1 Chittaranjan National Cancer Institute (CNCI), Kolkata, India; 2 All India Institute of Medical Sciences, New Delhi, India; 3 Department of Onco Radiology, BRAIRCH, All India Institute of Medical Sciences, New Delhi, India

**Keywords:** AI and ovarian cancer, artificial intelligence in ovarian cancer, diagnosis of ovarian cancer by artificial intelligence, machine learning in ovarian cancer, ML in ovarian carcinoma, ovarian carcinoma and AI

## Abstract

**Objective:**

To investigate a panel of variables using four machine learning based classifiers, i.e., support vector machine (SVM), random forest (RF), artificial neural network (ANN) and logistic regression (LR) to make a diagnosis of ovarian cancer, differentiating it from benign ovarian masses.

**Materials and methods:**

A prospective observational pilot study was done between November 2021 and June 2023. Following data pre-processing to ensure compatibility with ML models, four ML algorithms, i.e., support vector machine (SVM), logistic regression (LR), random forest (RF) and artificial neural network (ANN) were tested by multimodal parameters from the datasets of 50 patients presenting with suspected epithelial ovarian cancer (Group A) or benign ovarian tumour (Group B). Statistical analysis was done using STATA version 14.0.

**Results:**

We found that the machine learning approach could predict malignant tumours with appreciably high accuracy similar to a few studies done so far in this field. All four ML algorithms showed high level of accuracy with a maximum AUROC of 0.92 in the RF model. Both RF and SVM had an accuracy of 85.87 and 83.05%.

**Conclusion:**

The ML algorithms can detect ovarian cancers with a high level of accuracy. Further, a large-volume prospective study on large volume data sets is required before inclusion of ML algorithms in clinical practice.

## Introduction

Diagnosis of ovarian cancer often requires a multimodal approach like clinical history, examination, family history, radiological investigations, and blood biomarkers. The current standard of care for epithelial ovarian cancer (EOC) is either primary cytoreductive surgery or neo adjuvant chemotherapy (NACT) in an upfront setting (1). Even with the modern medical facilities, till date, ovarian cancer is considered as a silent killer, often presenting with nonspecific symptoms, normal tumour markers and overlapping morphological features on radiology, causing five year survival rates below 30% (2). The diagnostic conundrum in differentiating benign from malignant ovarian masses is real and often leads to over or undertreatment of adnexal masses in upfront setting, causing an impact on the survival of the patients suffering from this deadly disease. As a part of the pre operative investigation, radiological imaging, like ultrasonography (USG), computerized tomography (CT) scans, magnetic resonance imaging (MRI) and positron emission tomography (PET) CT scans have their own advantages and disadvantages in reaching to a conclusive diagnosis (3–5). As a result, patients presenting with ovarian mass often have to undergo multiple radiological investigations for decision making. Moreover, access to advanced imaging techniques like PET CT and MRI are frequently not feasible in resource limited settings.

In this scenario, the inclusion of artificial intelligence (AI) in preoperative characterization of ovarian masses and improving the accuracy of diagnosis would be valuable (6). In recent times, various machine learning (ML) algorithms have become quite entrenched in clinical cancer research and are often explored in clinical oncology services (7, 8). Inclusion of ML in preoperative triage would help in appropriate referral of patients for treatment. This is especially important in low and middle income countries (LMICs) where there is a lack of trained gynaecologic oncologists and dedicated cancer centres. The tested algorithms can be implemented in detection and management of ovarian cancer in a more objective way.

To the best of our knowledge, this is the first study in India where AI was evaluated to test the accuracy of radiological investigations such as USG, MRI, CT scan or PET CT in management of ovarian masses. The software was developed at the Centre for Development of Advanced Computing (C-DAC) Pune in collaboration with the All India Institute of Medical Sciences, Delhi. The objective of this pilot study was to investigate a panel of variables using four machine learning based classifiers, i.e., support vector machine (SVM), random forest (RF), artificial neural network (ANN) and logistic regression (LR) to make a diagnosis of ovarian cancer, and differentiating it from benign ovarian masses.

## Materials and methods

### Study population

A prospective observational pilot study was carried out from November 2021to June 2023. Ethical clearance was obtained from the institutional ethics committee (no IECPG637/28.10.21). Patients were broadly classified in two groups: Group A, in which patients aged ≥ 18 years with suspected epithelial ovarian cancer were invited to participate. Exclusion criteria were non-epithelial ovarian cancer, patients receiving neoadjuvant chemotherapy, borderline ovarian tumour, incomplete radiological and pathological records, and those unwilling to participate.

**Figure 1:**
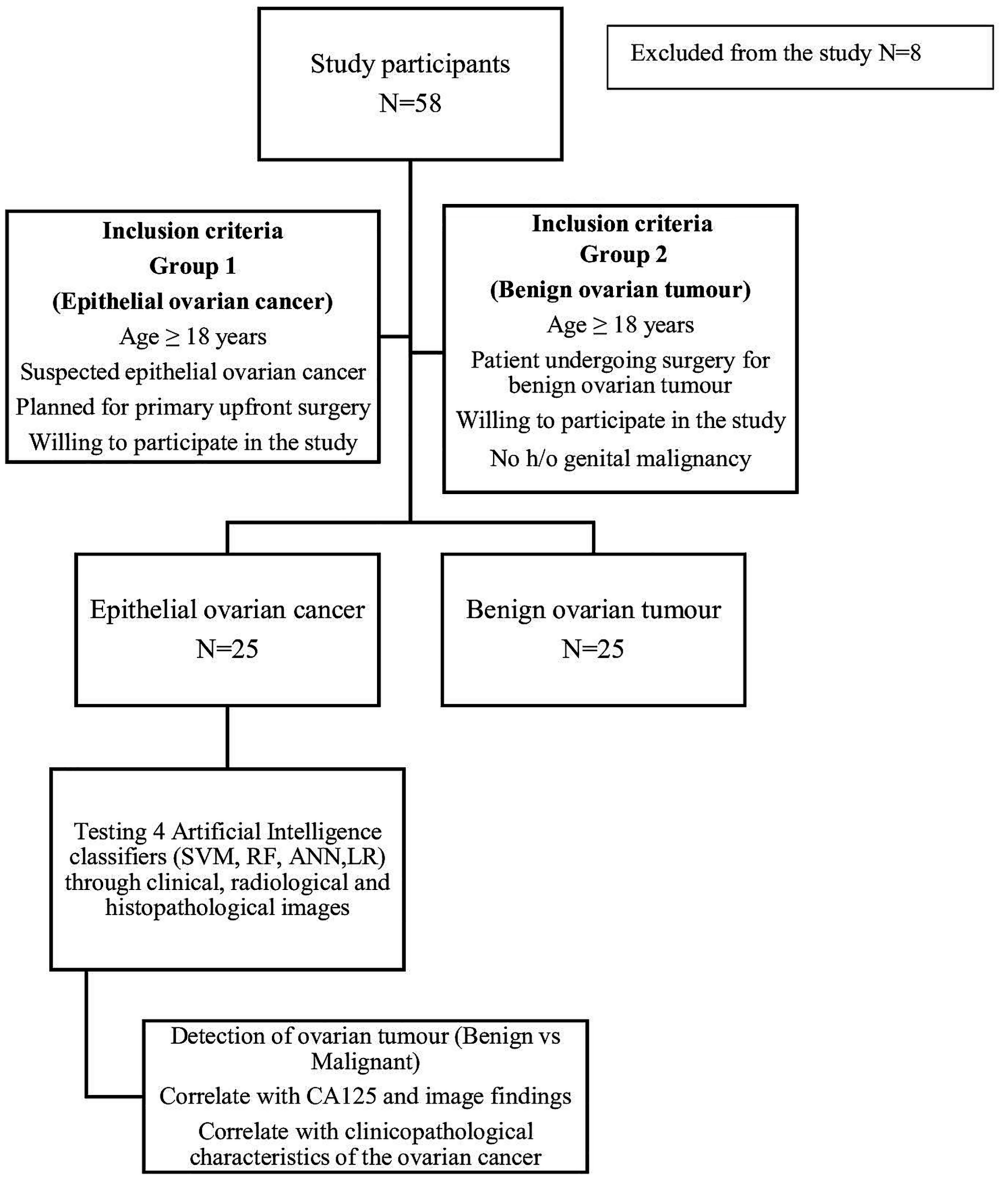
Study algorithm.

The inclusion criteria for Group B were age ≥ 18 years, undergoing surgery for benign ovarian tumour, willing to participate in the study and no history of genital malignancy. The exclusion criteria for this group were patients undergoing conservative or medical management for benign ovarian tumour and those unwilling to participate in the study. Sociodemographic characteristics were recorded, clinical examination and pre operative investigations were done as per hospital protocol. All patients underwent one or more of the following radiological investigations, e.g., ultrasonography (USG), CT scan whole abdomen, magnetic resonance imaging (MRI) or PET-CT. Patients with radiological investigations done elsewhere were included in the study if they could provide the imaging records. The morphological features were evaluated by MRI or USG. USG findings were recorded by O-RADS reporting system (9). In case a patient had multiple radiological investigations, the radiologist selected the best images which were then uploaded in the CVAT software. Histopathology was considered as the gold standard for final recruitment as per the inclusion and exclusion criteria of the study. The study algorithm is summarized in Figure 1.

Datasets were obtained from the clinical features, radiological images and histopathological images of all 50 patients who underwent upfront surgery in Group A and Group B. Once the radiological images were uploaded, the images were manually annotated through computer vision annotation tool (CVAT) software. The collected dataset comprised 20 features used to diagnose ovarian tumours as either benign or malignant, of which 5 were baseline characteristics, i.e., age, menopausal status, duration of symptoms, parity, and ECOG performance score, while 12 were radiological features, i.e., size, characteristics, laterality, locularity, ascites, papillary projections, septations, omental disease, retroperitoneal nodes, peritoneal metastasis and O-RADS score and 3 blood biomarkers (serum CA125, CEA, CA19.9). Additionally, histology images of the malignant and benign cases were uploaded for algorithm evaluation. The features were selected on the basis of their relevance in detection and management of EOC (10, 11).

### AI model training and evaluation

Data pre-processing was done to ensure compatibility with ML models and check for any inconsistencies. Recognizing the challenges of applying machine learning to a small oncology dataset, we adopted a cautious and clinically informed data splitting strategy. To test the developed models, the 50 patient dataset was split using the ‘train-test-split’ function. As the data was directly obtained from patients’ health records, pre-processing was done to handle inconsistencies and ensure compatibility with machine learning models. To address issues such as special symbols and incorrect values, we developed a class called remove special chars transformer. This class systematically identified and removed special symbols within the dataset, resulting in a clean dataset without such inconsistencies. There were no missing values recorded in the data set. To reduce the risk of overfitting, an important concern in small clinical cohorts, we deliberately restricted model complexity, applied regularization techniques, and monitored consistency across training and validation folds.

The voting selector algorithm (VSA) was used to improve the accuracy and reliability of feature subset identification by combining the outputs of multiple feature selection methods. The most suitable feature selection method was chosen by implementing and evaluating 11 different techniques. Out of these, three were filter methods, namely Mutual Information, ReliefF, and Monte Carlo; five were wrapper methods, namely, Forward Selection, Backward Selection, Boruta, Genetic Algorithm, and Simulated Annealing; lastly, three were embedded methods, namely, Recursive Feature Elimination, Ecological Model Rule Forest (EMRF), and Ecological Model Logistic Regression (EMLR). The VSA was applied and evaluated on Group A and Group B dataset to assess its performance in identifying informative feature subsets by the consensus voting process and maximize the model’s performance by selecting the hyperparameters. The fine tuning of the hyperparameter was performed using 5-fold cross-validation for four different algorithms: support vector machine (SVM), logistic regression (LR), random forest (RF) and artificial neural network (ANN). For the ANN algorithm, additional multiple layers of framework were tested. The best hyperparameters and their corresponding scores were then recorded. The GridSearchCV object performed a systematic search over this parameter grid using individual algorithm as the estimator. A total 3,000 plus image data sets were annotated including histology images of both benign and malignant ovarian tumours.

To test the developed models, the 50 patient dataset was split using the ‘train-test-split’ function.

Once the best hyperparameters for each model were obtained, the next step was to evaluate and compare the performance of the models on the classification task. Evaluation was based on the accuracy score, precision, recall and F1 score for measurement of functionality of each ML algorithm. The AUC (Area Under the Curve) measured the overall performance of the model across all thresholds. To derive a consensus feature selector algorithm, we combined the top four algorithms and considered the features selected by most of these algorithms. Thus the most effective model for the given classification task was identified. Finally, the ensemble learning method was used as a hard voting scheme with the top four models using the voting classifier function of sklearn library. Alongside traditional ML algorithms in a relatively limited dataset, additionally, we tested 2 deep learning algorithms ResNet50 (Residual Neural Network) and Inception Net v3 (GoogleNet) for bench marking and comparison with 4 ML algorithms. As the manuscript focused on the 4 ML algorithms, the details of the DL models were beyond the discussion in this manuscript.

After continuous epoch of all four ML algorithms, the best value was chosen for the each algorithm.

#### Management of the ovarian masses

All patients had upfront surgery according to standard of care for the disease type (12). For cancer cases, the peritoneal carcinomatosis index (PCI) score was calculated intraoperatively, and cytoreduction (CC) score was documented as CC0, CC1, CC2 or CC3. The histopathologic subtype of the tumour and grade was classified according to the World Health Organization (WHO) 2020 guidelines for tumours of female reproductive organs (13). The final stage was assigned according to the International Federation of Gynecology and Obstetrics (FIGO) 2014 staging for carcinoma ovary (14). Benign ovarian tumours were managed as per standard of care.

#### Statistical analysis

Data were censored on June 20, 2023. Data analysis was carried out using statistical software STATA version 14.0. Continuous variables were tested for normality assumptions using the Kolmogorov–Smirnov test. Descriptive measures such as mean, standard deviation (SD) and range values were calculated for normally distributed data. Comparison of mean values between the groups was performed using Students‘t-independent test/ANOVA as appropriate. For ML evaluation, variance threshold, Spearman correlation, and Z statistic test were applied to remove irrelevant features and perform feature selection.

## Results

### Sociodemographic characteristics

A total of 58 women were invited to participate in the study: Group A comprised of 30 women with epithelial ovarian cancer, while Group B comprised of 28 women with benign ovarian tumours. Eight patients had to be excluded for the following reasons: final histology leiomyoma (*n* = 1) and borderline ovarian tumour (*n* = 2), CD images not available (*n* = 4), withdrew consent (*n* = 1). Thus, 50 patients were included in the final analysis, with 25 women in each group. The sociodemographic characteristics of women participated in the study are summarized in Table 1.

**Table 1:** Baseline sociodemographic characteristics.

Socio-demographic characteristics	Categories	Group A (malignant)	Group B (benign)	*p* value
Age (years)	18–24	0 (0%)	6 (24%)	**0.028**
	25–34	3 (12%)	7 (28%)	
	35–44	6 (24%)	4 (16%)	
	45–54	7 (28%)	5 (20%)	
	>55	12 (36%)	3 (12%)	
Education	No formal education	4 (16%)	0 (0%)	0.096
	Primary- Middle school	5 (20%)	6 (24%)	
	High school	8 (32%)	5 (20%)	
	College	8(32%)	14 (56%)	
Occupation	Student	0 (0%)	5 (20%)	0.054
	Home maker	17 (68%)	11(44%)	
	Service/Business	8 (32%)	9 (36%)	
Marital status	Married	24 (96%)	16(64%)	**0.011**
	Unmarried	1 (4%)	9(36%)	
	Widowed	0 (0%)	0 (0%)	
Parity	0–1	4 (16%)	14 (56%)	0.015
	2 to 3	15 (60%)	8(32%)	
	>3	6 (24%)	3 (12%)	
Socioeconomic status	Upper	4 (16%)	3 (12%)	0.839
	Upper middle	9 (36%)	7 (28%)	
	Lower middle	7 (28%)	10(40%)	
	Upper lower	4 (16%)	5 (20%)	
	Lower	1 (4%)	0 (0%)	

Bold value indicates socio economic status (modified kuppuswamy scale).

The mean age of presentation in Group A was 49.60 ± 12.21 years and in Group B was 36.56 ± 14.95 years. The majority of women in both the groups had a ECOG ACRIN PS of 1 (56% vs. 84% in Group A and B respectively). The median duration of symptoms was 56 days (range 21–224 days) in Group A and 120 days (range 30–365 days) in Group B, with the most common presenting symptoms being distension of abdomen (44%) and mass per abdomen (40%), respectively. Among the risk factors, age and menopausal status were statistically significant (*p* < 0.05), whereas hormone intake, body mass index and family history of cancer were not statistically significant. Table 2 shows the radiological characteristics in both groups of patients.

**Table 2:** Radiological characteristics.

Radiological findings	Categories	Group A (malignant)	Group B (benign)	*p* value
Size	≤10 cm	10 (40%)	6 (24%)	0.364
	>10 cm	15 (60%)	19 (76%)	
Characteristics	Solid	5 (20%)	2 (8%)	**0.003**
	Cystic	3 (12%)	19 (76%)	
	Solid cystic	17 (68%)	4 (16%)	
Laterality	Unilateral	14 (56%)	24 (96%)	**0.002**
	Bilateral	11 (44%)	1 (4%)	
Papillary projections	Single	2 (8%)	1 (4%)	**0.001**
	Diffuse	9 (36%)	0	
	None	14 (56%)	24 (96%)	
Locularity	Unilocular	11 (44%)	19 (76%)	**0.042**
	Multilocular	14 (56%)	6 (24%)	
Septations	≤ 3 mm	13 (52%)	20 (80%)	0.072
	>3 mm	12 (48%)	5 (20%)	
Retroperitoneal nodes	Present	5 (20%)	1 (4%)	0.189
	Absent	20 (80%)	24 (96%)	
Ascites	Present	6 (24%)	0	**0.022**
	Absent	19 (76%)	25 (100%)	
Omental disease	Present	10 (40%)	0	**0.001**
	Absent	15 (60%)	25 (100%)	
Peritoneal metastases	Present	9 (36%)	0	**0.002**
	Absent	16 (64%)	25 (100%)	
Colour score	0–2	16 (64%)	22 (88%)	0.095
	3 to 5	9 (36%)	3 (12%)	
O-RADS (USG/MRI)	0 to 1	3 (12%)	1 (4%)	**0.007**
	2 to 3	6 (24%)	17 (68%)	
	4 to 5	16 (64%)	7 (28%)	

Bold values indicates socio economic status (modified kuppuswamy scale).

Intraoperatively, the mean PCI value of the cancer cases was 9.64 ± 7.14. In 92% of cases, complete cytoreduction (CC) 0–1 was achieved. In 2 (8%) women, >2.5 cm residual disease had to be left behind because of peritoneal carcinomatosis. Out of 25 carcinoma ovary cases, 52% (*n* = 13) patients were of HGSOC, 24% (*n* = 6) LGSOC, 16% (*n* = 4) were mucinous carcinoma, and rest were endometrioid and clear cell carcinoma. For the benign counterpart, 40% (*n* = 10) were serous cystadenoma, 28% (*n* = 7) were mucinous cystadenoma, 20% (*n* = 4) endometrioma and 12% (*n* = 3) mature cystic teratoma. There was one case of serous cystadeno-fibroma on final histology. Adjuvant treatment for ovarian cancer cases were given according to the stage of the disease and following institutional protocol.

### Performance of the machine learning algorithms

**Figure 2:**
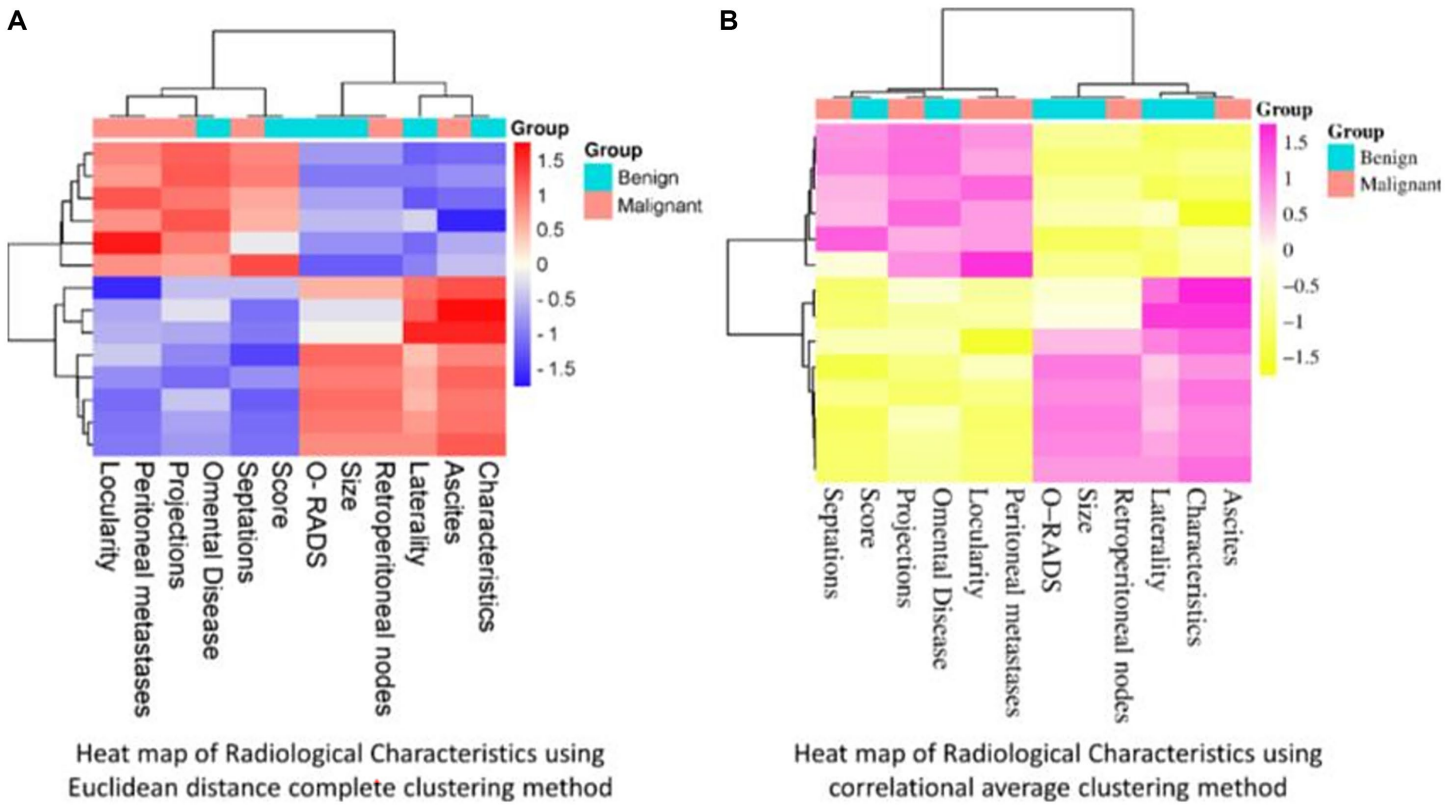
**(A,B)** Heat map of radiological characteristics of ovarian tumours according to Euclidean distance complete clustering method and correlational average clustering method.

Figures 2A,B show the heat map describing the correlation between different independent radiological variables using Euclidean distance and average clustering method.

As shown in Figure 2A, the radiological findings namely, locularity, peritoneal metastases, papillary projections, septations and ascites were highly significant (1.5) for Group A, i.e., malignant cases; while colour score, O-RADS, size, laterality, and characteristics of the tumour were crucial in Group B, i.e., benign cases. Retroperitoneal nodes were more significant in malignant cases whereas *p* value was not significant when measured in standard logistic regression. Figure 2B shows that when average linkage clustering was used, radiological characteristics, namely, septations, peritoneal metastases and ascites were highly significant and were co-dependent (1.5) for Group A while, O-RADS, size, laterality, and characteristics were correlated (−1) features in Group B cases.

### Diagnostic accuracy of AI algorithms

Table 3 shows the various performance metrics of all 4 ML algorithms.

**Table 3:** Performance metrics for the ML algorithms.

AI model	Accuracy	Precision	Recall	F1-score	AUROC
RF	0.84	0.82	0.86	0.84	0.92
SVM	0.88	0.85	0.93	0.88	0.90
LR	0.84	0.79	0.90	0.85	0.90
ANN	0.81	0.83	0.79	0.80	0.82
ResNet50	0.99	0.98	0.98	0.98	0.99
InceptionNet v3	0.98	0.98	0.98	0.98	0.99

All four ML algorithms showed high level of accuracy with maximum AUROC of 0.92 in RF model. Based on the crucial characteristics, RF and SVM were best performing when compared to counterparts LR and ANN. Both RF and SVM had an accuracy of 85.87 and 83.05%, respectively. For the DL set, both ResNet50 and Inception Net v3 had highest AUROC 0f 0.99.

**Figure 3:**
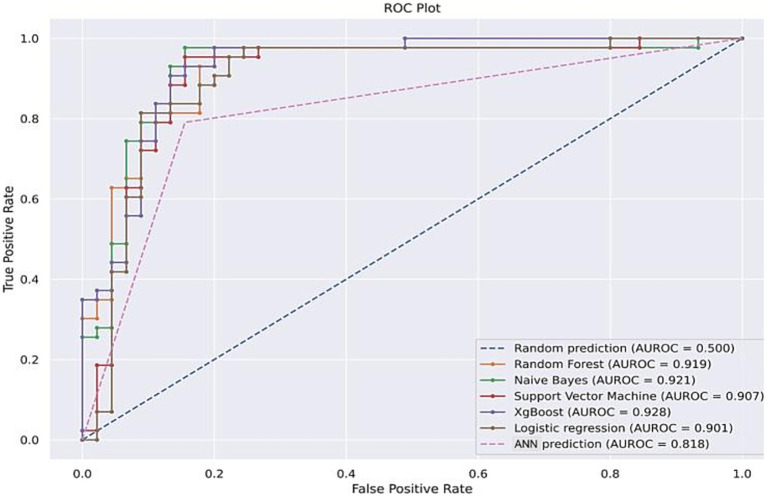
ROC curve of ML models used on testing dataset.

Figure 3 depicts the ROC performances for all the ML algorithms.

The ROC curve was plotted to detect the sensitivity and specificity of all 4 ML algorithms. In addition, XG boost and Naïve Bayes were included for benchmarking and comparison with RF, LR, ANN and SVM. Out of all 4 ML algorithms, RF outperformed the other 3 algorithms with an AUROC of 0.92.

## Discussion

The current standard of care in ovarian cancer prognostication and treatment decisions are based on clinical parameters, multiple imaging, biomarkers, etc., The complexities of identification and advance management of ovarian cancer are increasing with the availability of novel biomarkers and targeted therapies (15–18). For a practicing clinician, it is challenging to keep pace with the rapid advancement in this area.

The traditional management of any adnexal mass is surgery (19). This study is the first from India where artificial intelligence has been evaluated for its role in preoperative detection of ovarian cancer and to differentiate between benign and malignant ovarian tumours. The correct identification of the cancer and appropriate referral to a Gynaecological Oncologist saves time and resources which is often difficult by the clinicians with limited resources and high volume of patients attending their clinic. To address this challenge and taking the help of AI, the evaluation of multiple performance metrics of the four ML algorithms allowed us to gain a comprehensive understanding of each algorithm’s strengths and weaknesses. During the model training, the F1 score combined precision and recall into a single metric, and was especially useful as our dataset number was small (*n* = 50), and accuracy alone may not accurately represent the model’s effectiveness. This study is instrumental in showing us the path where clinicians can take help of the machine to handle symptomatic ovarian masses in a more smarter and accurate way.

Most of the studies done so far, have included blood parameters and other physical characteristics to test ML algorithms and shown that ML technology was superior to conventional logistic regression in predicting EOC diagnosis (20–23).

A study done by Eiryo Kawakami et al. in 2019, where 334 patients with EOC and 101 patients with benign ovarian tumours were randomly assigned to “training” and “test” cohorts (24). Seven supervised machine learning classifiers, including gradient boosting machine (GBM), support vector machine, random forest (RF), conditional RF (CRF), naïve Bayes, neural network, and elastic net, were used to derive diagnostic and prognostic information from 32 parameters commonly available from pre-treatment peripheral blood tests and age. The values for the highest accuracy and area under the ROC curve (AUC) for segregating EOC from benign ovarian tumours with RF were 92.4% and 0.968, respectively. They concluded that machine learning systems could provide a critical diagnostic and prognostic prediction for patients with EOC before the initial intervention, and the use of predictive algorithms may facilitate personalized treatment options through pre-treatment stratification of patients.

Our study is similar to the study done by Akazawa and Hashimoto (25). A total 202 patients with ovarian tumours were enrolled, including 53 with ovarian cancer, 23 with borderline malignant tumours, and 126 with benign ovarian tumours. Using five machine learning classifiers, including support vector machine, random forest, naive Bayes, logistic regression, and XGBoost, they derived diagnostic results from 16 features, commonly available from blood tests, patient background, and imaging tests. Their study observed promising results of AI in the prediction of pathological diagnosis of ovarian cancer from preoperative examinations. We primarily focused on the radiological parameters and tested the algorithms along with tumour markers and physical characteristics. There was a higher level of diagnostic accuracy than the conventional logistic regression in preoperative diagnosis of ovarian cancer. In this study RF had the highest accuracy with AUROC 0.92 followed by SVM and LR with similar accuracy (AUROC 0.90) ANN had the least diagnostic accuracy in the detection of ovarian cancer with AUROC 0.82. The highest accuracy with DL models may help us to build more complex algorithms in near future, but at present beyond the scope of discussion in the present manuscript.

The strengths of the study were its prospective design and the clinical attempt to evaluate artificial ML models for preoperative diagnosis of ovarian cancer and to differentiate between benign and malignant ovarian tumours. It is the first prospective pilot study in India wherein we tested the algorithms by using the objective parameters of radiological investigations. This makes the study more robust instead of using non-specific blood parameters. Approximately 3,000 image data sets were analysed which gave a robust impression of diagnostic accuracy of ML algorithms.

The limitations of the study were the less number of subjects in view of time constraints. More data sets need to be tested by the supervised algorithm before the information can be utilised in clinical practice.

However, this pilot study is based on single dataset, but this prepares the base of testing the artificial intelligence in large- volume data sets with encouraging results. A prospective multi institutional study on the similar lines to externally validate the models in larger cohorts in future will help to better assess their generalizability and support translation into real-world clinical oncology practice. This may be the answer for many logistic and technical challenges we faced during the study.

## Conclusion

We found that the machine learning approach could predict malignant tumours with appreciably high accuracy similar to a few studies done so far in this field. All four ML algorithms, i.e., random forest, support vector machine, logistic regression, and artificial neural network performed well in the detection of ovarian cancer with the highest level of accuracy shown by the RF model. Artificial intelligence can help the decision-making of surgeons in challenging cases with atypical findings in preoperative examinations, and therefore, early and accurate diagnosis of ovarian cancer could lead to an improved prognosis. AI can predict the definitive diagnosis by combining the results of preoperative examinations and producing the numerical value of the probability of ovarian cancers, the management of ovarian tumours could be significantly improved and therefore it could be explained more accurately to patients and their relatives. This is more relevant in low and middle income countries like India where AI can help in accurate diagnosis of the disease and help the physicians to refer accordingly. It prepares the base for testing the artificial intelligence in large volume data sets with encouraging results. A larger prospective study on similar lines may be the answer for many logistic and technical challenges we faced during the study. This could establish the place of AI in facilitating personalized treatment options through promising pre-treatment stratification of EOC patients.

## Data Availability

The raw data supporting the conclusions of this article will be made available by the authors, without undue reservation.
